# Enhanced Proapoptotic Effects of Water Dispersed Complexes of 4-Thiazolidinone-Based Chemotherapeutics with a PEG-Containing Polymeric Nanocarrier

**DOI:** 10.1186/s11671-019-2945-7

**Published:** 2019-04-23

**Authors:** L. Kobylinska, I. Ivasechko, N. Skorokhyd, R. Panchuk, A. Riabtseva, N. Mitina, A. Zaichenko, R. Lesyk, B. Zimenkovsky, R. Stoika, S. G. Vari

**Affiliations:** 1 Department of Biochemistry, Danylo Halytsky Lviv National Medical University, Pekarska St., 69a, Lviv, 79010 Ukraine; 2 Department of Pharmaceutical, Organic and Bioorganic Chemistry, Danylo Halytsky Lviv National Medical University, Pekarska St., 69a, Lviv, 79010 Ukraine; 3 Department of Regulation of Cell Proliferation and Apoptosis, Institute of Cell Biology, Drahomanov St., 14/16, Lviv, 79005 Ukraine; 4 Department of Organic Chemistry, Lviv Polytechnic National University, S. Bandera., 12, Lviv, 79013 Ukraine; 5 International Research and Innovation in Medicine Program, Cedars-Sinai Medical Center, Los Angeles, CA 90048-5502 USA

**Keywords:** 4-thiazolidinones, Polyethylene glycol, Polymeric nanocarrier, Apoptosis, Rat glioma C6 cells

## Abstract

**Aim:**

To study whether water formulation of the complex of 4-thiazolidinone derivatives with a PEG-containing polymeric nanocarrier enhances their pro-apoptotic action towards rat glioma C6 cells.

**Methods:**

Mechanisms of antineoplastic effects of 4-thiazolidinone derivatives were investigated in vitro with rat glioma C6 cells. Cell nativity, cell cycling pattern, and Annexin V expression were evaluated and DNA damage was estimated by DNA comet analysis. A novel water-based formulation of 4-thiazolidinone derivatives complexed with a polymeric nanocarrier was utilized for enhancing pro-apoptotic action towards C6 cells.

**Results:**

The studied 4-thiazolidinone derivatives use apoptosis mechanisms for killing rat glioma C6 cells, as confirmed by FACS analysis of these cells in pre-G1 stage, the appearance of Annexin V positive C6 cells, and an increased number of DNA comets of higher classes. Complexation of the studied compounds with a PEG-containing polymeric nanocarrier significantly increased pro-apoptotic effects in rat glioma C6 cells measured by all methods mentioned above.

**Conclusion:**

Complexation of 4-thiazolidinone derivatives with a PEG-containing polymeric nanocarrier provided them with water solubility and enhanced pro-apoptotic effects in rat glioma C6 cells.

## Introduction

Chemotherapy is the main treatment modality for many tumors, and chemotherapeutic drugs are chosen as the last option, especially in cases when cancer has already spread [1]. However, the severe negative side effects reduce the clinical efficacy of many current anticancer drugs. Therefore, there is a continual and crucial need to develop alternative or synergistic anticancer drugs with reduced or minimal side effects [2].

The low water solubility of anticancer drugs has been a prevalent and serious issue that hinders their development and clinical application, and many highly active and promising chemotherapeutic agents have been excluded because of their low water solubility [3]. Surfactants and co-solvents are used in high concentrations in order to solubilize water-insoluble anticancer agents; however, these substances can also produce adverse side effects, thus, limiting the use of many anticancer drugs [4].

A big challenge for the development of pharmaceutical drugs is to eliminate or at least reduce the negative side effects of highly active drugs, especially the antitumor agents demonstrating general toxicity in the body that significantly restricts their use [5–9]. An effective way to overcome this problem is to use a multifunctional nanocarrier of the drug that will allow the toxic antitumor agents to bind at the site of their delivery to targeted cells in specific organs or tissues [8, 10, 11]. The small size, controlled-specific structure, large surface area, and shape of nanoparticles provide them with several advantages compared to other materials [5, 8, 9]. When using nanoparticles, it is possible to optimize efficiency, minimize negative side effects, and improve cancer therapy [12, 13]. Thus, drugs that failed earlier due to high general toxicity can now get a second opportunity for clinical use, due to their inclusion in a delivery system that has improved bioavailability and controlled release. Such drug-carrier complexes more easily penetrate through natural barriers like blood–brain barriers or membranes of individual cells. The large surface area of nanoparticles makes it possible for them to form complexes with specific vector biomolecules that assist in drug delivery to the target organ, lead to a significant reduction or even complete elimination of negative side effects, allow an increase in the minimum effective dose during a single administration of the medicine, enhance the effectiveness of drug action, and give a new stimulus for the development of personalized pharmacotherapy. In addition, nanoparticles can encapsulate molecules that improve solubility, stability, and absorption of certain drugs [11, 13–15]. Drug delivery systems can provide prolonged circulation of a drug in the blood, the ability for the drug to accumulate during the pathophysiological process, and the capability of effectively transferring the molecule of the active substance into the cells and organelles of the cells. The nanoparticles must be stable, maintain their chemical structure for a certain time period, and at the same time, be capable of bio-degradation [16, 17].

To evaluate new anticancer drugs, it is important to measure their cytotoxic activity and potency using various target human cancer cell lines, and experimental tumor models in vivo*.* Chemotherapy often fails because of a deficiency in the apoptosis process that plays a pivotal role in drug-induced cell death consecutive to or resulting from a change in tumorigenesis [18–21].

Since many malignant cells can evade apoptotic death, a rational approach should be used in the design and development of new anticancer drugs. The major goals for creating new anticancer drugs are to (1) find ways to overcome mutations of individual cancer cells that impact independent mechanisms of drug action; and (2) design chemotherapy regimens capable of simultaneously targeting independent pathways. A better understanding of the relationship between cancer genetics and treatment sensitivity is a key issue for developing new effective anticancer drugs [22].

In previous studies, we demonstrated that synthetic 4-thiazolidinone derivatives (Les-3288, Les-3833, and Les-3882) probably use different mechanisms of action than other anticancer agents to kill rat C6 glioma and human U251 glioblastoma cells in vitro, contrary to doxorubicin (Dox). Les-3288 did not significantly affect the level of reactive oxygen species (ROS) in the treated cells [23, 24]. It should be stressed that these potent antitumor agents showed less general toxicity in the body of experimental animals, as demonstrated by the measured biochemical parameters of their toxic action in tumor cells and animals, compared with those of Dox [7, 8]. Thus, the binding of an antitumor drug with a polymeric nanocarrier (PNC) and drug application in the form of a stable water delivery system can reduce the toxic effects in the organs of animals, compared with the action of these substances in a free form [7, 8].

The aim of this work was to study apoptosis induction in rat glioma cells of the C6 line in vitro and in vivo by water-based formulations of complexes of 4-thiazolidinone derivatives with a PEG-containing PNC, and compare the apoptosis induction using these derivatives in free form.

## Materials and Methods

### Anticancer Drugs

**Fig. 1:**
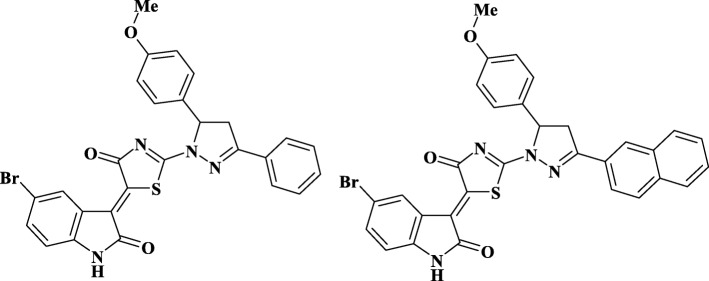
Structure of the investigated compounds—Les-3288 and Les-3833

The heterocyclic 4-thiazolidinones derivatives (compounds Les-3288 and Les-3833, Fig.1) were synthesized at the Department of Pharmaceutical, Organic and Bioorganic Chemistry of Danylo Halytsky Lviv National Medical University, Ukraine, as previously described [25].

Before use in cell culture, these compounds were dissolved in dimethyl sulfoxide (DMSO, Arterium, Lviv, Ukraine). The solution was additionally kept for 5 min in a boiling water bath, and diluted in distilled water in order to reach the working concentrations. The final concentration of the DMSO in culture medium was below 0.1%. Dox was bought in a local pharmacy from a Pfizer (Italy) representative in Ukraine.

### Polymeric Nanocarrier

**Fig. 2:**
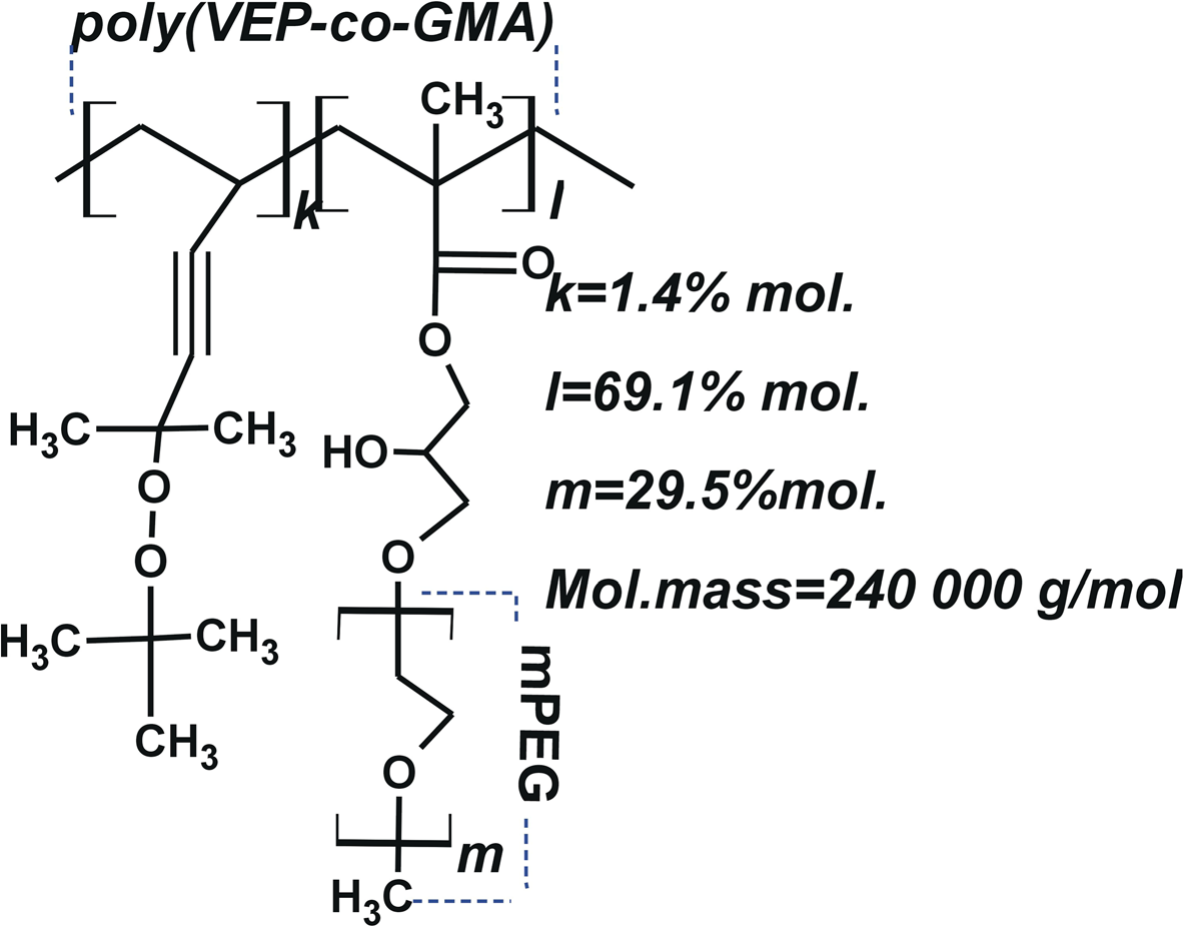
Schematic structure of the PNC — poly(VEP*-co-*GMA)-*graft*-PEG with the main chain on the base of a co-polymer of unsaturated peroxide of 2-*tert*-butylperoxy-2-methyl-5-hexen-3-in (VEP, denoted by “k”), with side chains of glycidyl methacrylate (GMA, denoted by “l”) and polyethylene glycole (PEG, denoted by “m”)

The PNC for drug delivery was synthesized at the Department of Organic Chemistry of Lviv Polytechnic National University, Ukraine, using a methodology described earlier [26, 27]. Synthesis of poly(VEP-*co*-GMA)-graft-PEG was carried out via subsequent stages as follows. Poly(VEP-*co*-GMA) was synthesized by radical copolymerization of 5-*tert* butylperoxy-5-methyl-l-hexene-3-yne (VEP, 0.41 g, 0.5 mol) (peroxide monomer synthesized by the described method [28]) and glycidyl methacrylate (GMA, 7.72 g, 12.2 mol) (Sigma-Aldrich, USA) in ethyl acetate (7.9 mL) (Merck, Darmstadt, Germany) using azoisobutyronitrile (AIBN, 0.129 g, 0.05 mol) (Merck, Darmstadt, Germany) as the radical initiator. Polymerization was carried out at 343K until the maximal conversion of 65% was reached. Poly(VEP-*co*-GMA) was used as the backbone for the attachment of side PEG chains via addition reactions of poly(ethylene glycol) methyl ether (mPEG) with side epoxide groups. Boron trifluoride etherate (0.027 mL, 0.031 mol) (Sigma-Aldrich, St. Louis, MO, USA) was added to the solution of copolymer (1.0 g) in dioxane (20 mL; Merck, Darmstadt, Germany) at 313 K and stirred for 3 h. mPEG (2.5 mg, 3.35 mmol) (Sigma-Aldrich, USA) was dissolved in dioxane (15 mL) and the solution was mixed with the solution of backbone polymer and stirred for 6 h at 313 K. Unreacted mPEG was removed using dialysis bags with pore size of molecular weight cut-off (MWCO) of 6-8 kDa (Sigma-Aldrich, USA). Resulting comb-like poly(VEP-*co*-GMA)-graft-PEG was dried at room temperature under vacuum until constant weight. The structures and some characteristics of the PNC are presented in Fig. 2 and were previously described [29].

To prepare the water-based PNC solutions, 0.09 g of PNC was dissolved in 0.9 mL DMSO. This solution of PNC was added to 8.0 mL of 0.9% NaCl solution. Then, the solution was stirred for 0.5 h and sonicated for 10 s. Water dispersions of the complexes were obtained by dropping the DMSO solution of 4-thiazolidinones and PNC into the water in the following order: 0.045 g of PNC was dissolved in 0.15 mL of DMSO (Sigma-Aldrich, USA), and 0.0015 g of Les-3288 (or Les-3833) was dissolved in 0.10 mL of DMSO. The solutions of PNC and 4-thiazolidinones were mixed and added to 4.25 mL of 1% aqueous NaCl solution and sonicated for 10 s.

This surface-active PNC contains the hydrophilic PEG chains grafted to the hydrophobic backbone (Fig. 2a). In aqueous solutions, the amphiphilic PNC forms micelle-like structures that can solubilize water-insoluble compounds (e.g., drugs) or these compounds can be adsorbed onto the surface of the nanoparticles, thereby increasing their biocompatibility. The technique developed for obtaining highly stable water dispersions of the 4-thiazolidinone-based chemotherapeutics consists of the nucleation of nanoscale drug particles that occurs when the diluted solutions in DMSO are transferred into the precipitant saline solution containing polymeric surfactant PNC that provides polymer coating for the nanoparticle surface. As a result, due to aggregation and sedimentation, the complex was stabilized and the dispersion was prevented. In addition, the nanoscale complexes of 4-thiazolidinone derivatives are protected from possible interactions with the surrounding biological microenvironment. This protection allows them remaining longer in the body without losing stability and accumulate in the target tissue or organ without causing negative side effects. Thus, such modification provides enhanced aggregation and sedimentation stability of water dispersions of the anticancer compound nanoparticles.

**Fig. 3:**
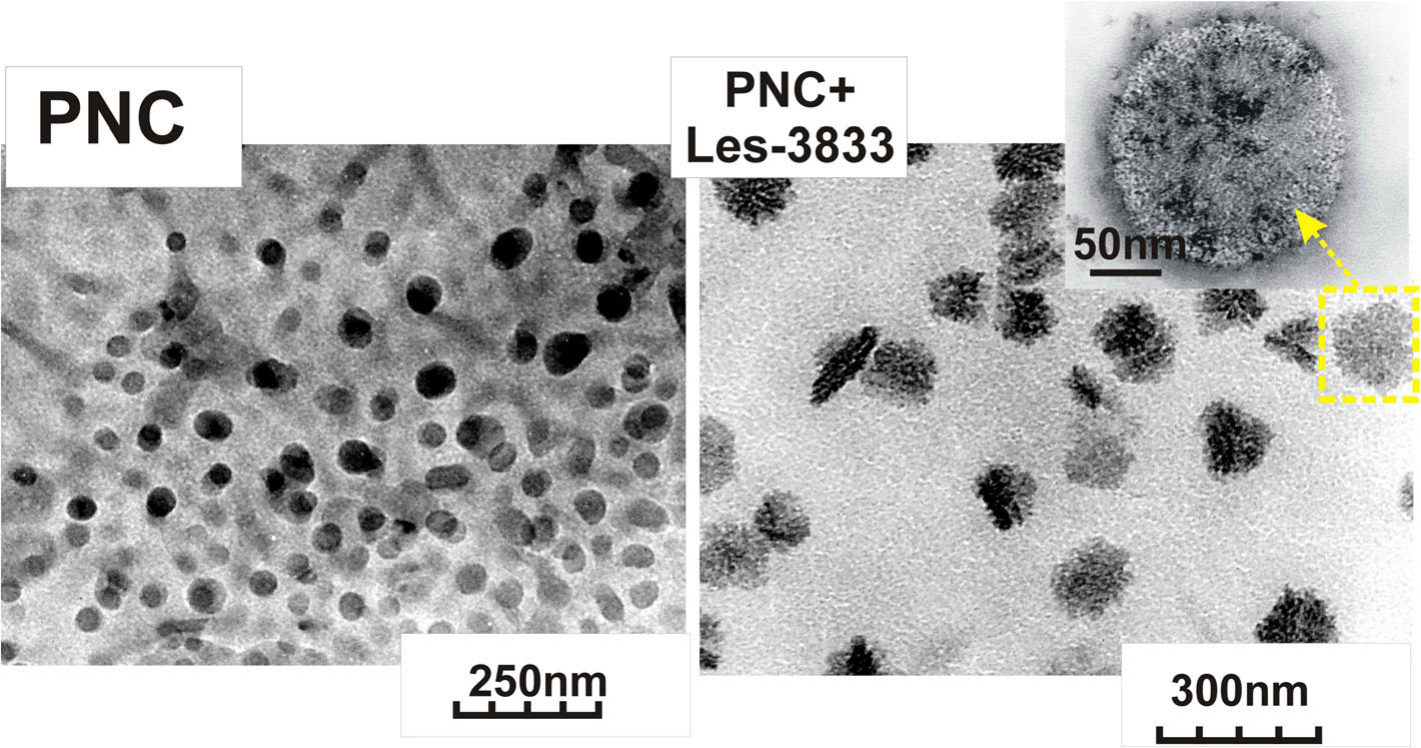
Transmission electron microscopy (TEM) images of the PNC (left) and complex of PNC + Les-3833 (right)

The sizes of the polymer micelles of PNC and of the complexes of PNC with heterocyclic 4-thiazolidinones were measured by dynamic light scattering (DLS) using a Zetasizer Nano ZS (Malvern Instruments GmbH, Stuttgart, Germany) and DynaPro NanoStar (Wyatt Technology, Santa Barbara, CA, USA) and by photon correlation spectra using non-invasive back scatter (NIBS) technology at 25°C. The samples for DLS measurements were prepared as described above, and if necessary, were diluted with bi-distilled water, pH 6.5–7.0. Three-five measurements were made for every sample (each measurement consisted of 5 cycles and the range between measurements was 5 min). Figure 3 shows the size distribution of micellar structures of the PNC and the nanoparticles of the complexes of PNC with the heterocyclic 4-thiazolidinones (Les-3833 and Les-3288) analyzed by DLS. The size and morphology of PNC micelles as well as of PNC-coated 4-thiazolidinone nanoparticles were studied using a transmission electron microscope JEM-200А (JEOL, Japan) at an accelerating voltage of 200 kV [30, 31]. The samples were prepared as described above, and if necessary, were diluted with bi-distilled water. Samples were prepared by spraying the tested solution onto a substrate by means of the ultrasonic dispersant UZDN-1A (Ukrrospribor Ltd., Ukraine), which facilitates a uniform coating on the substrate. A thin amorphous carbon film deposited on a copper grid was used as a substrate. The sizes and morphology of PNC micelles and the complexes with the heterocyclic 4-thiazolidinones (Les-3288 and Les-3833) studied by the methods of DLS and TEM are presented in Fig. 3.

**Fig. 4:**
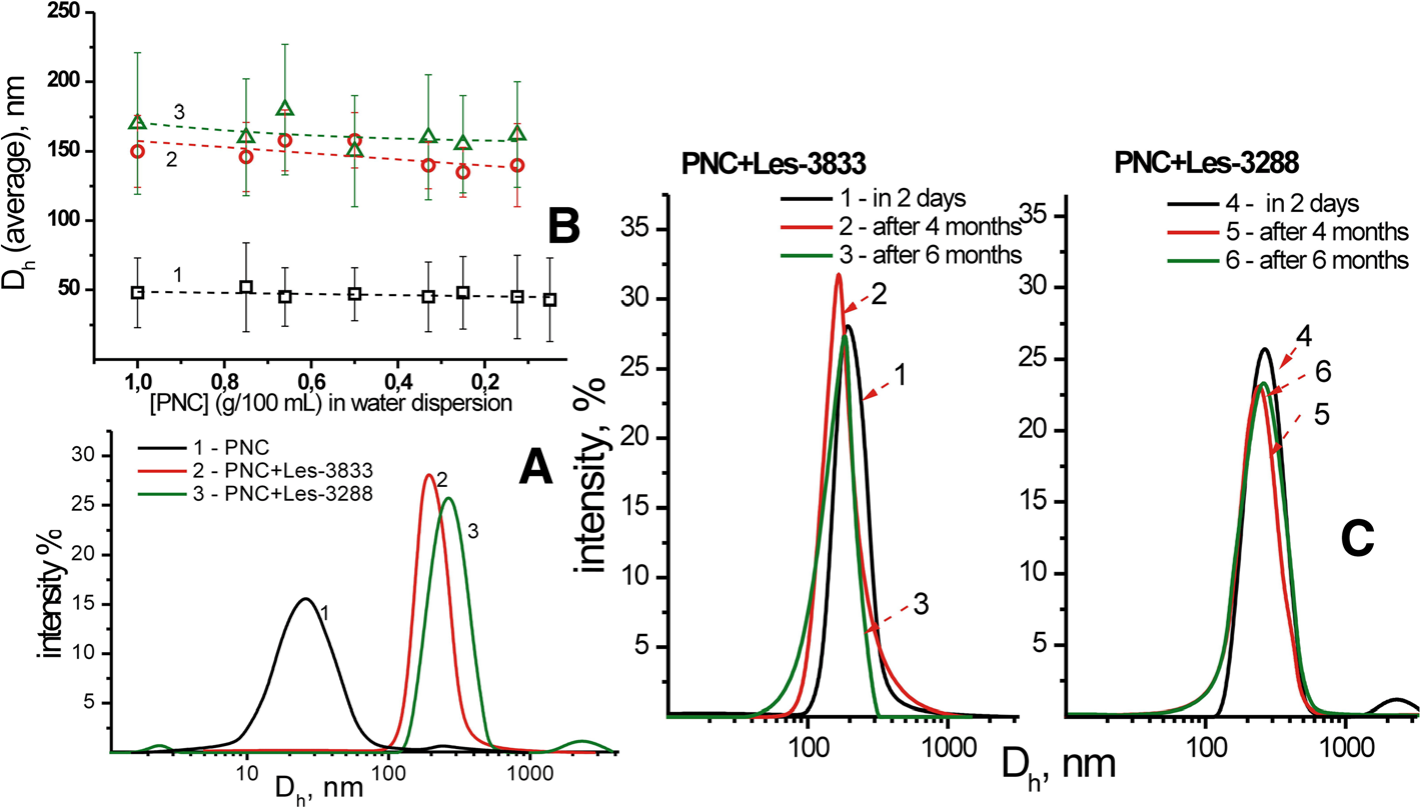
DLS study of hydrodynamic diameters of PNC and complexes (**a**) and dependence of average sizes of PNC-drug complexes on dispersion dilution (**b**): water dispersions of PNC (1), complexes of Les-3833+PNC (2), and Les-3288+PNC (3), as well as hydrodynamic sizes of Les-3833+PNC (1–3) and Les-3288+PNC (4–6) dispersions after storage for 2 days (1.4), 4 months (2.5), and 6 months (3.6) (**c**)

The DLS and transmission electron microscopy (TEM) studies demonstrated that the sizes of PNC micelles were 50 ± 25 nm and the nanoparticles formed by complexes of PNC with the heterocyclic 4-thiazolidinones Les-3833 and Les-3288 were 140 ± 25 nm and 155 ± 30 nm, respectively*.* The dispersions of complexes of the PNC with 4-thiazolidinone derivatives are highly stable and protected from aggregation and sedimentation by the adsorbed PNC shell on the thiazolidinone nanoparticle surface. As shown in Fig. 4, changes in sizes of the nanoparticles dispersed in the water system are negligible at multiple dilutions with water, as well as after 6 months of aging of the water systems of complexes of PNC with 4-thiazolidinones.

### Cell Culture

Rat glioma cells of the C6 line were obtained from Cell Culture Collection at the Institute of Molecular Biology and Genetics, National Academy of Science of Ukraine (Kyiv, Ukraine). Cells were cultured in Dulbecco’s modified Eagle’s medium (DMEM, Sigma, USA) supplemented with 10% fetal bovine serum (Sigma, USA). Cells were grown in a CO_2_-incubator at 37°C, 5% CO_2_, and 95% humidity. The reseeding of cells was carried out at a ratio of 1:5 once in 2–3 days.

### Evaluation of Cytotoxic Action of Studied Substances

Cells were seeded in 24-well plates at a concentration of 100,000 cells/mL in one well. (Greiner Bio-One North America, Inc., Monroe, NC, USA). The substances under study were added at 0.1, 0.5, and 1.0 μM concentrations after a 24 h adaptation period that started after cell seeding. Cell counting was conducted at regular intervals in the hemocytometric counting chamber using Trypan Blue dye (DV-T10282, Invitrogen, Thermo Fisher Scientific Corp., Waltham, MA, USA) at 0.01% final concentration, 2 min after its addition to the cell suspension. The dead cells took up this dye due to damage to their plasma membrane.

### Flow Cytometry

For studying cell cycling and apoptosis, C6 cells were treated with Les-3288, Les-3833 and their complexes with PNC, as well as with PNC in free form, washed with phosphate-buffered saline (PBS, pH 7.4), pelleted by centrifugation at 1000 rpm for 5 min at 4°C and re-suspended in cold PBS (2 million cells per 1 mL of PBS). Then, cells were fixed by adding aliquots of 4 mL of absolute ethanol cooled to − 20°C with gentle mixing. The fixed samples were kept at − 20°C until use (no longer than 1 week). For fluorescence-activated cell sorting (FACS) analysis, cell samples were centrifuged at 1000 rpm for 5 min at 4°C, the supernatant was discarded and the cell pellet was re-suspended in 1 mL of PBS. One hundred microliters of DNase-free RNase (Sigma, USA) was added to that suspension, and samples were incubated at 37 °C for 30 min. Then, each sample was supplemented with 100 μl of propidium iodide (PI) (Sigma, USA, 1 mg/mL), and incubated at room temperature for 10 min. Finally, the samples were transferred to plastic Falcon tubes, and the cell suspension was monitored with a FACSCalibur flow cytometer (BD Biosciences, Mountain View, CA, USA) and Summit v3.1 software (Cytomation, Inc., Fort Collins, CO, USA) for measuring the parameters of cell cycling and apoptosis.

Measurement of the Annexin V-positive (apoptotic) cells was performed using FACS analysis. Rat glioma C6 cells were treated with 0.1 mg/mL, 0.5 mg/mL, and 1 mg/mL concentrations of PNC, Les-3288, Les-3833, and their polymeric conjugates as indicated under the figures. At the end of the experiment, the cells were detached from the bottom of the dish with Trypsin-EDTA solution, washed twice with PBS, and stained with Annexin V-FITC using Apoptosis Detection Kit (BD Biosciences, San Jose, CA, USA), according to the manufacturer’s instructions. Washed cells were incubated for 15 min in the Annexin V binding buffer (BD Pharmingen), containing 1/50 volume of FITC-conjugated Annexin V solution. Then, the samples were diluted twice with an appropriate volume of the Annexin V binding buffer and immediately measured on the FL1/FL2 (FITC-PI) channel of a flow cytometer (Becton Dickinson, USA). The Annexin V-single positive cells were classified as apoptotic.

### DNA Comet Assay

The DNA comet assay was carried out under alkaline conditions. Ten thousand cells were mixed in 75 μl of low melting point agarose (0.5%) per sample. After mixing, the sample was pipetted onto a slide that had been covered with 1.5% normal melting point agarose beforehand. The samples were incubated at 4 °C during 18 h in lysis solution (2.5 M NaCl, 100 mM EDTA, 10 mM Tris-base, 10% DMSO, 1% Triton X-100). To allow unwinding of the DNA and the expression of alkali-labile damage, slides were incubated in an alkali solution at room temperature for 20 min (conducted in dark). For electrophoresis (~74 V/cm for 30 min), slides were transferred to a horizontal chamber and 1x electrophoresis buffer (0.3 M NaOH, 1 mM EDTA, pH > 13) was added. The slides were fixed in ice-cold, 100% methanol and dried. Slides were stained with 80 μL 1× ethidium bromide (EtBr) for 5 min and then dipped in chilled distilled water to remove excess stain. The DNA comets were visualized using a microscope (Carl Zeiss, Germany), and the images were analyzed using the software ‘CASP’ (Casplab-1.2.3b2 software, CASPlab, Wroclaw, Poland). One hundred comets per sample were counted. DNA damage was categorized into five levels of genotoxicity according to the comet tail size: 0 (0–5% damage), 1 (5–25% damage), 2 (25–45% damage), 3 (45–70% damage), and 4 (more than 70% damage) [32]. The damage index (DI) was calculated, as previously described [33].

### DNA Intercalation Assay Using Methyl Green Dye

The compounds Les-3288 and Les-3833 were investigated for the ability to intercalate into the DNA molecule by using methyl green assay [34]. Salmon sperm DNA (10 mg/mL) was incubated for 1 h at 37 °C with 15 μL of methyl green solution (1 mg/mL in H_2_O). The compounds were added at 1 μg/mL and incubated at 37 °C in the dark for 2 h. The total final volume of the samples was 1 mL. Absorption of methyl green was measured at 630 nm, after sample incubation for 2 h at 37 °C, using a BioTek 76 883 multi-channel microphotometer (BioTek, Winooski, VT, USA). EtBr was used as a positive control.

### Data Analysis and Statistics

All experiments were repeated three times with three parallels in each variant. Analysis of variance (ANOVA) was used for comparison of groups. Data of the distribution of C6 rat glioma cells in different phases of the cell cycle were analyzed by one-way ANOVA, followed by Tukey’s post hoc multiple comparison test. All data are presented as a mean ± SD. A *p* value of < 0.05 was considered as statistically significant.

## Results

### Cytotoxic Effect (Trypan Blue Exclusion Test) of 4-Thiazolidinone Derivatives Used in Free Form and in Complex With the Polymeric Nanocarrier

**Fig. 5:**
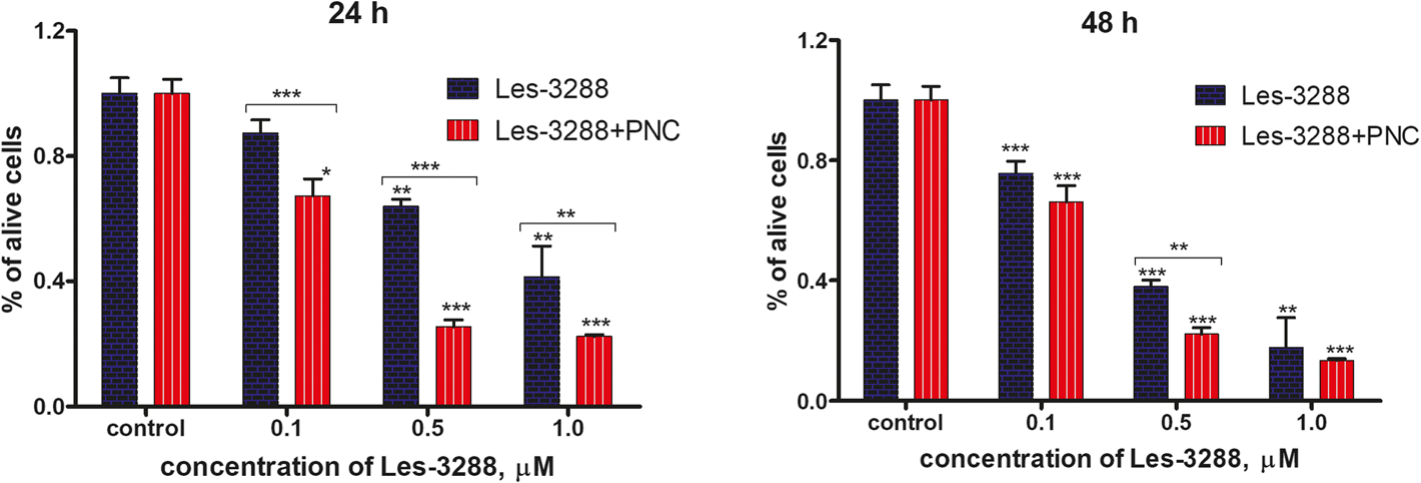
Number of living rat glioma C6 cells measured by Trypan blue exclusion test after treatment for 24 h and 48 h with different concentrations (0.1, 0.5, and 1.0 μM) of Les-3288 alone compared with the action of its complex with the polymeric nanocarrier (PNC) *P < 0.05; **P < 0.01; ***P < 0.001 *(*difference in comparison to the control, 100%)

**Fig. 6:**
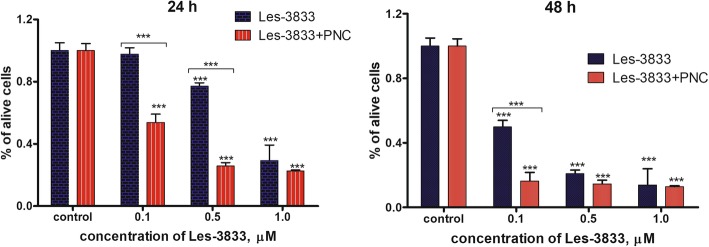
Number of living rat glioma C6 cells measured by Trypan blue exclusion test after treatment for 24 h and 48 h with different concentrations (0.1, 0.5, and 1.0 μM) of Les-3833 alone compared with the action of its complex with the polymeric nanocarrier (PNC) *P < 0.05; **P < 0.01; ***P < 0.001 (difference in comparison to the control, 100%)

The results of the Trypan blue exclusion test demonstrated that the water-based forms of Les-3288 and Les-3833 complexes with the PNC had enhanced toxicity towards glioma C6 cells (24 h and 48 h treatment) compared with the cytotoxicity observed from using the free form of these compounds (Figs. 5 and 6). The highest cytotoxicity effect was observed at 0.1 and 0.5 μM doses of Les-3288 and Les-3833 compounds in their complexes with the PNC, while at the highest dose (1.0 μM) of these compounds, an enhancement of complexation with the PNC was detected only in the case of Les-3288 for 24 h (Figs. 5 and 6).

### Complexation of Les-3833 and Les-3288 Compounds With Polymeric Nanocarrier Enhances Their Pro-Apoptotic Activity In Vitro Towards Rat Glioma C6 Cells

Two alternative approaches were used to assess the modulatory effect of the PNC on the cytotoxic potential of the selected 4-thiazolidinone derivatives. First, cell cycle analysis was conducted to investigate the impact of the drug-PNC complexes on cell cycling. Additionally, Annexin V/PI double staining was utilized to distinguish the exact type of cell death induced by these nanocomposites.

**Fig. 7:**
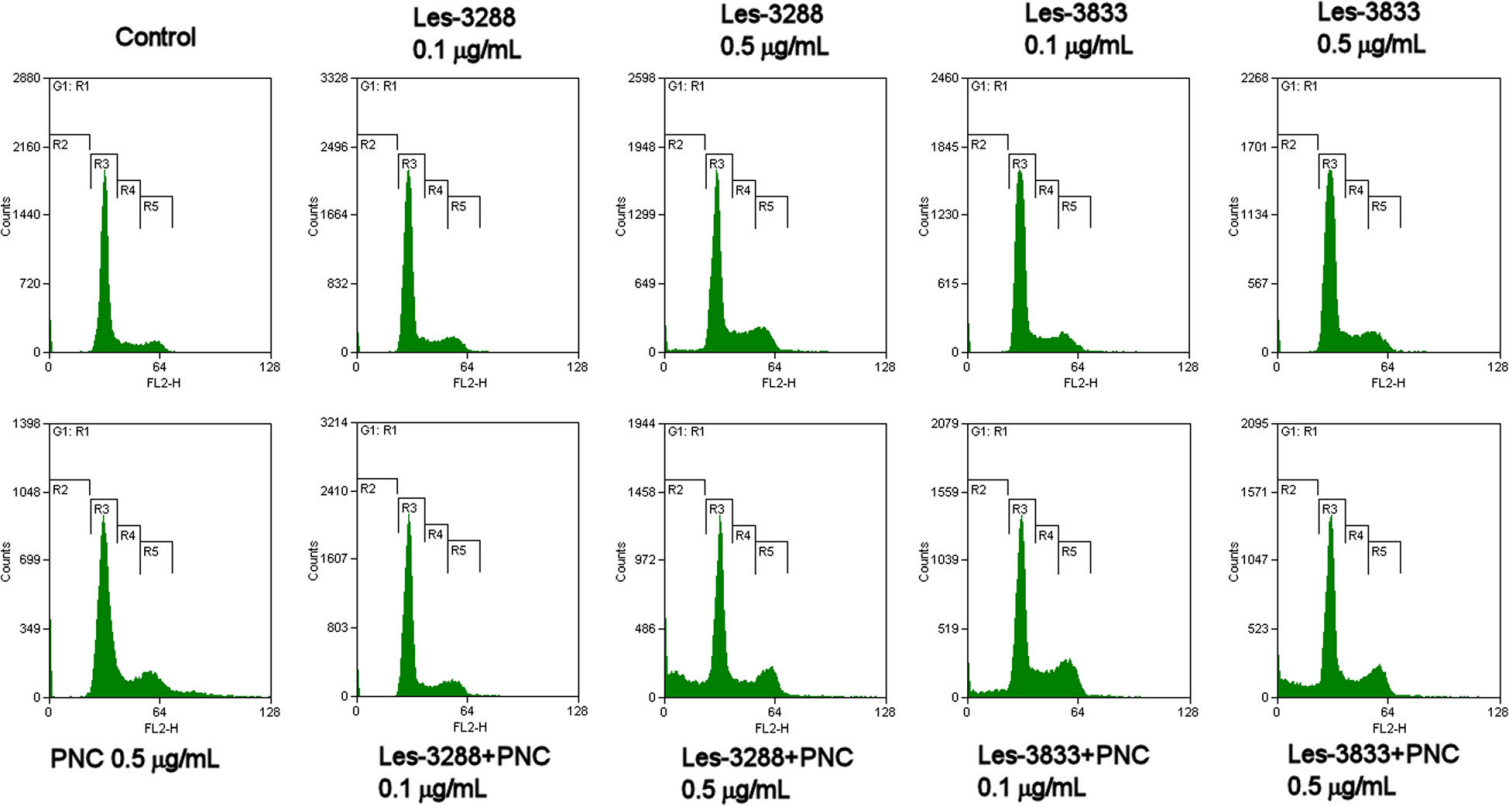
Impact on cell cycle progression of 4-thiazolidinone derivatives Les-3288 and Les-3833 in free form and in complex with the polymeric nanocarrier (PNC) in C6 rat glioma cells. Propidium iodide (PI) staining, flow cytometry. R2, pre-G1; R3, G1; R4, S; R5, G2 phase. FL2-H, peak emission values of the second channel (585/40 filter) of flow cytometer

Les-3288 and Les-3833 compounds at low doses (0.1 μg/mL and 0.5 μg/mL) produced no visible effect on either cell cycle progression or induction of apoptosis, as shown in Fig. 7 and Table 1. However, complexation of both compounds with the PNC significantly increased the number of cells in pre-G_1_ phase (4-fold for Les-3288 and 6-fold for Les-3833), which indicates a major boost of their pro-apoptotic potential. It should be stressed that PNC in free form had no impact on cell cycling or apoptosis induction; thus, the observed appearance of a large population of pre-G1 cells under treatment with the thiazolidinone-nanocarrier complexes cannot be explained by a simple synergistic effect of the PNC and the experimental drugs.

**Table 1:** Distribution of C6 rat glioma cells in different phases of the cell cycle under treatment for 24 h with 4-thiazolidinone derivatives Les-3288 and Les-3833 in free form and in complex with polymeric nanocarrier (PNC)

Sample	Number of cells in each cell cycle phase, mean ± SD			
	Pre-*G*_1_	*G*_1_	*S*	G_2_/*M*
Control	3.52 ± 0.67	75.06 ± 0.57	11.61 ± 0.22	10.82 ± 1.21
PNC 0.1 μg/mL	3.30 ± 0.44	76.03 ± 0.52	13.33 ± 0.41	8.81 ± 0.92
PNC 0.5 μg/mL	5.78 ± 0.51	61.30 ± 0.50^***^	12.07 ± 0.12	16.06 ± 0.18
Les-3288 0.1 μg/mL	2.81 ± 0.45	75.99 ± 0.06	12.01 ± 0.43	10.32 ± 1.10
Les-3288 0.5 μg/mL	5.99 ± 0.54^*^	63.33 ± 0.67^***^	16.09 ± 0.12^***^	15.19 ± 0.43^***^
Les-3288 + PNC 0.1 μg/mL	3.27 ± 0.31	73.54 ± 0.39	12.48 ± 1.20	11.44 ± 0.40
Les-3288 + PNC 0.5 μg/mL	24.89 ± 1.49^●●●^	47.65 ± 0.34^●●●^	9.77 ± 0.53^●●●^	16.45 ± 0.16
Les-3833 0.1 μg/mL	3.06 ± 0.45	72.99 ± 0.84^*^	12.92 ± 1.11	12.07 ± 0.31
Les-3833 0.5 μg/mL	2.88 ± 0.67	72.53 ± 1.09^**^	13.23 ± 0.56	12.64 ± 0.41
Les-3833+PNC 0.1 μg/mL	9.11 ± 0.53^###^	54.86 ± 0.57^###^	17.40 ± 0.61	19.55 ± 1.41^#^
Les-3833+PNC 0.5 μg/mL	18.69 ± 1.71^§§§^	53.42 ± 0.21^§§§^	12.30 ± 0.55	15.32 ± 1.91

Data are presented as mean ± SD

*P < 0.05 related to control, **P < 0.01 related to control, ***P < 0.001 related to control, ^●●●^*P* < 0.001 related to Les-3288, 0.5 μg/mL, ^#^*P* < 0.01 related to Les-3833, 0.1 μg/mL, ^###^*P* < 0.001 related to Les-3833, 0.1 μg/mL, ^§^*P* < 0.05 related to Les-3833, 0.5 mg/mL, ^§§§^*P* < 0.001 related to Les-3833, 0.5 mg/mL

**Fig. 8:**
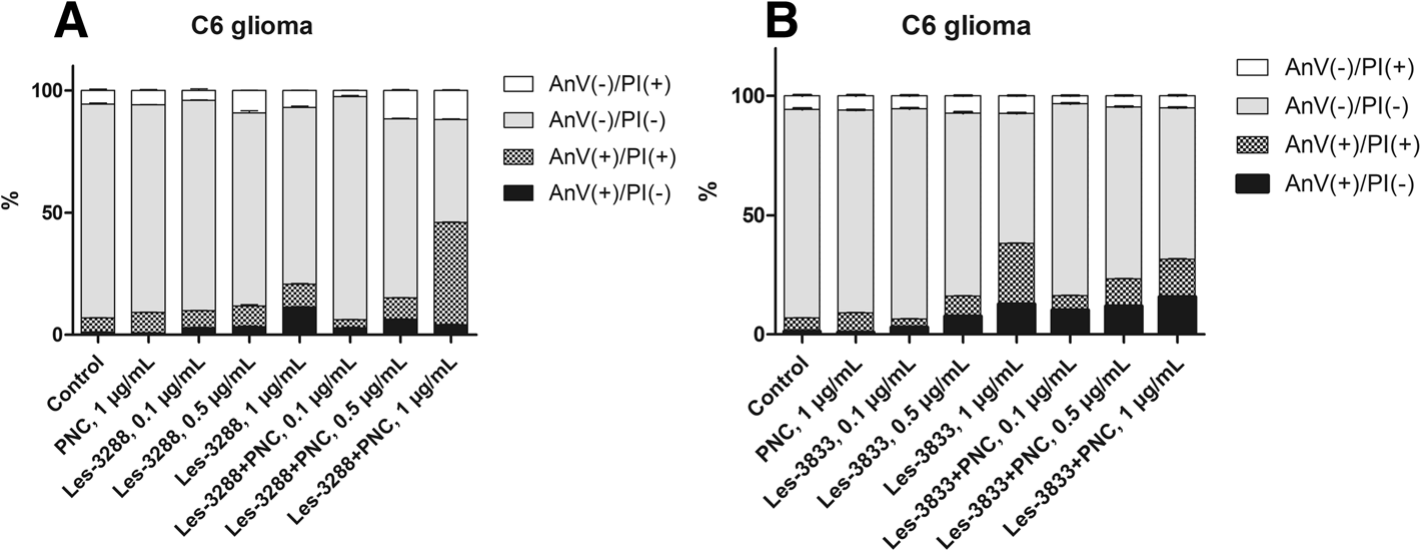
Comparison of the pro-apoptotic activity of Les-3288 (**a**) and Les-3833 (**b**) compounds and their complexes with the polymeric nanocarrier (PNC) towards rat glioma C6 cells. Annexin V/PI double staining, flow cytometry. PI, propidium iodide

In order to confirm that the observed sub-G1 peak consists of the apoptotic cells, the results of the Annexin V/PI staining assay were analyzed. We did not observed significant externalization of phosphatidylserine (main marker of early apoptosis, detected by FITC-conjugated Annexin V) during C6 glioma treatment with low (0.1 and 0.5 μg/mL) and high doses of Les-3288 (1.0 μg/mL), suggesting that this compound is a weak inducer of apoptosis. Instead, we found an increase in the necrotic cell population (from 1.2% in control to 11.25% for Les-3288, 1.0 μg/mL), which may indicate that Les-3288 at least partially causes to necrotic cell death. However, complexation of Les-3288 with the PNC completely changed the mode of action of this drug. There was a massive increase in the numbers of early apoptotic (Annexin V(+)/PI(−)) and late apoptotic cells (Annexin V (+)/PI(+)), while the overall quantity of purely necrotic cells remained at the level of control (untreated) cells (Fig. 8a). Thus, binding Les-3288 with the PNC not only strongly enhances its pro-apoptotic activity towards glioma cells, but also decreases its potential pro-necrotic cell death mechanism. These findings may be of key importance for clinical practice because the pro-necrotic drugs are not recommended for use due to a massive induction of inflammation which is inconvenient for patients.

Surprisingly, we did not observe the same effect for the Les-3833+PNC complex. In fact, the pro-apoptotic activity of the Les-3833+PNC complex was lower compared to Les-3288 in free form at all tested concentrations (Fig. 8b). Such a drastic difference between both compounds might be explained by specificity of their chemical structure, and we speculate that in the case of Les-3833, the structure of the polymer should be optimized to achieve better results.

### Comet Assay of DNA Damage in Rat Glioma C6 Cells Caused by 4-Thiazolidinone Derivatives in Free Form and Complexed With the PNC

**Fig. 9:**
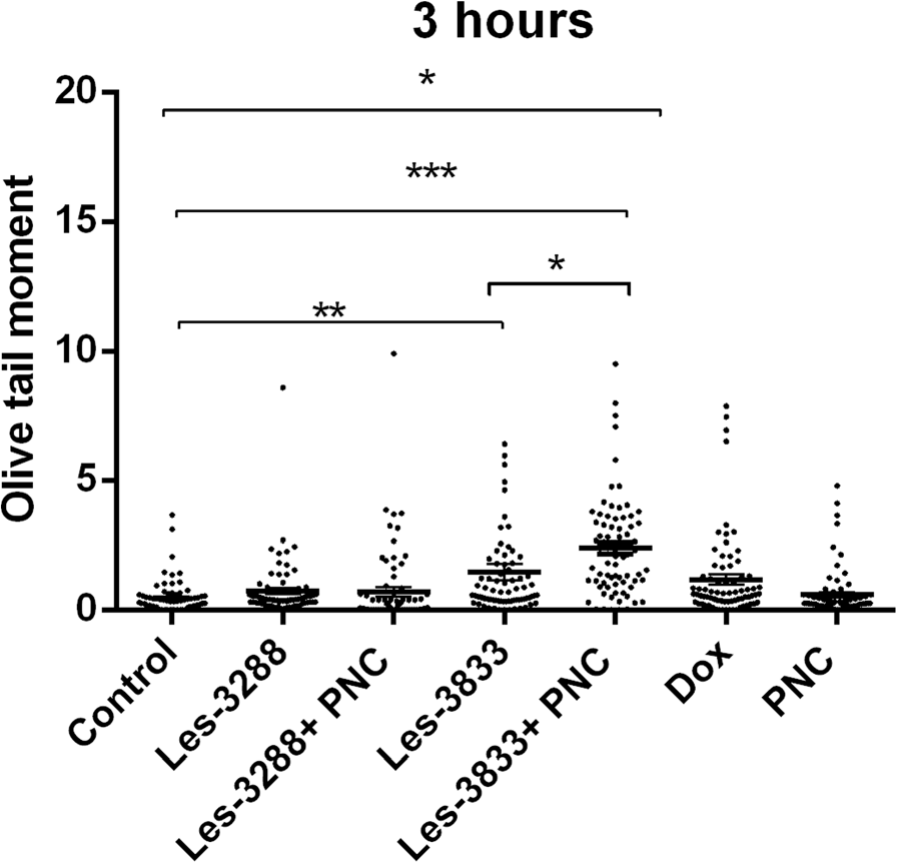
DNA damage was evaluated using the Olive tail moment in rat glioma C6 cells treated for 3 h with experimental antineoplastic compounds used at a 0.5 μg/mL concentration. *P ≤ 0.05; **P ≤ 0.01; ***P ≤ 0.001. PNC, polymeric nanocarrier; Dox, doxorubicin (positive control)

**Fig. 10.**
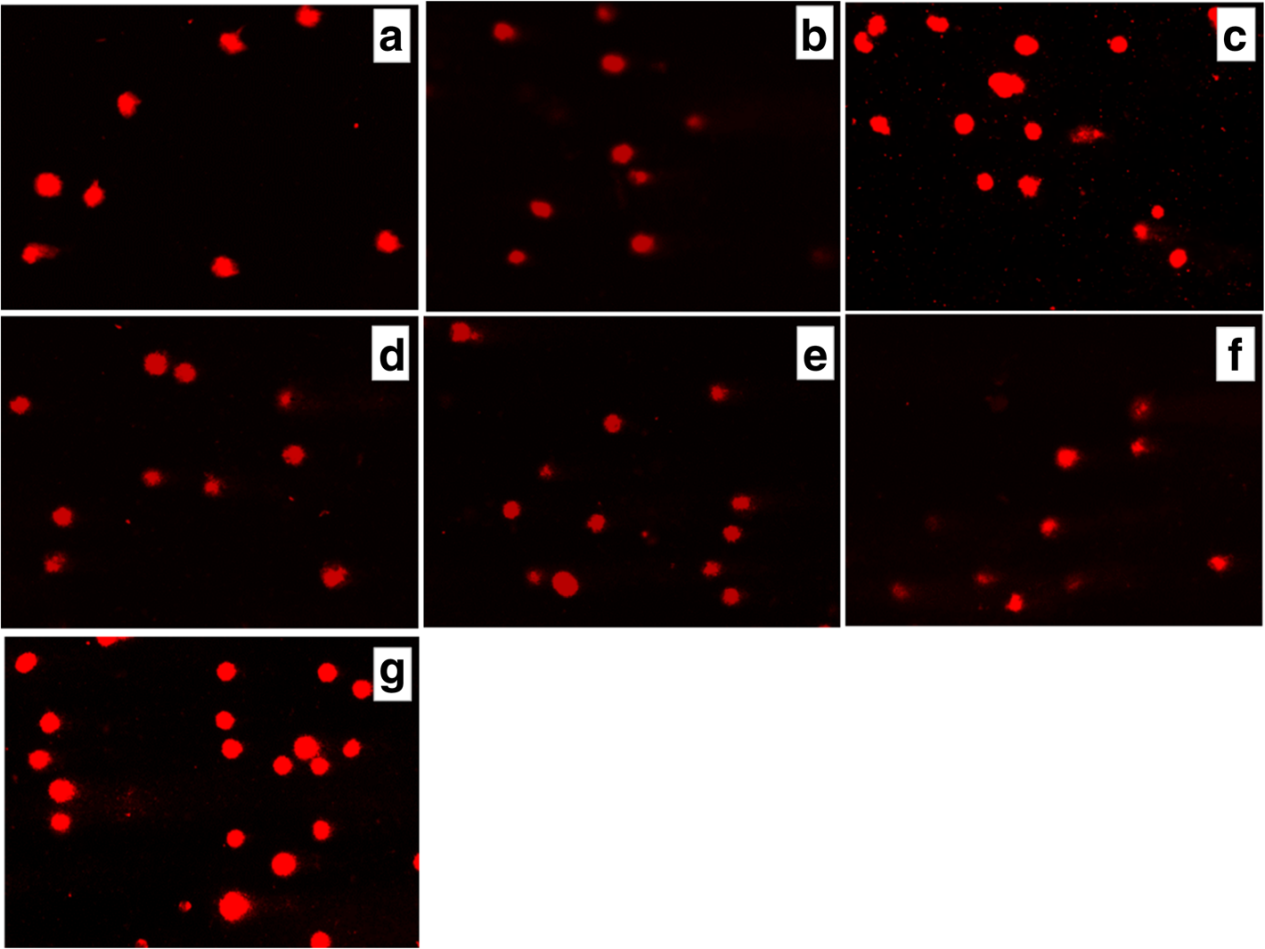
Presence of DNA in comet tail of rat glioma С6 cells after treatment for 3 h with (**a**) control (untreated cells), Les-3288 (**b**), Les-3288+PNC (**c**), Les-3833 (**d**) Les-3833 + PNC (**e**), PNC (**f**) and doxorubicin (**g**), used at a 0.5 μg/mL concentration

Alkaline comet assay allows the detection of single-strand DNA breaks in alkaline-labile DNA sites. The obtained results were estimated using the mean value of the Olive tail moment (OTM). Our data showed that treatment of С6 rat glioma cells with Les-3288, Les-3833, and PNC (concentration 0.5 μg/mL) for 3 h (Figs. 9 and 10) did not cause a significant DNA damage (OTM = 0.7299 ± 0.1276, OTM = 1.466 ± 0.3086, OTM = 0.5846 ± 0.1078, respectively), compared with the untreated cells in the control (OTM = 0.4541 ± 0.07273). However, cell treatment with the Les-3833 + PNC complex (OTM = 2.3880 ± 0.2212) caused more significant DNA damage than cell treatment with the free form of Les-3833. Such an increase in damage was not observed for the action of the Les-3288+PNC complex (Figs. 9 and 10).

**Fig. 11:**
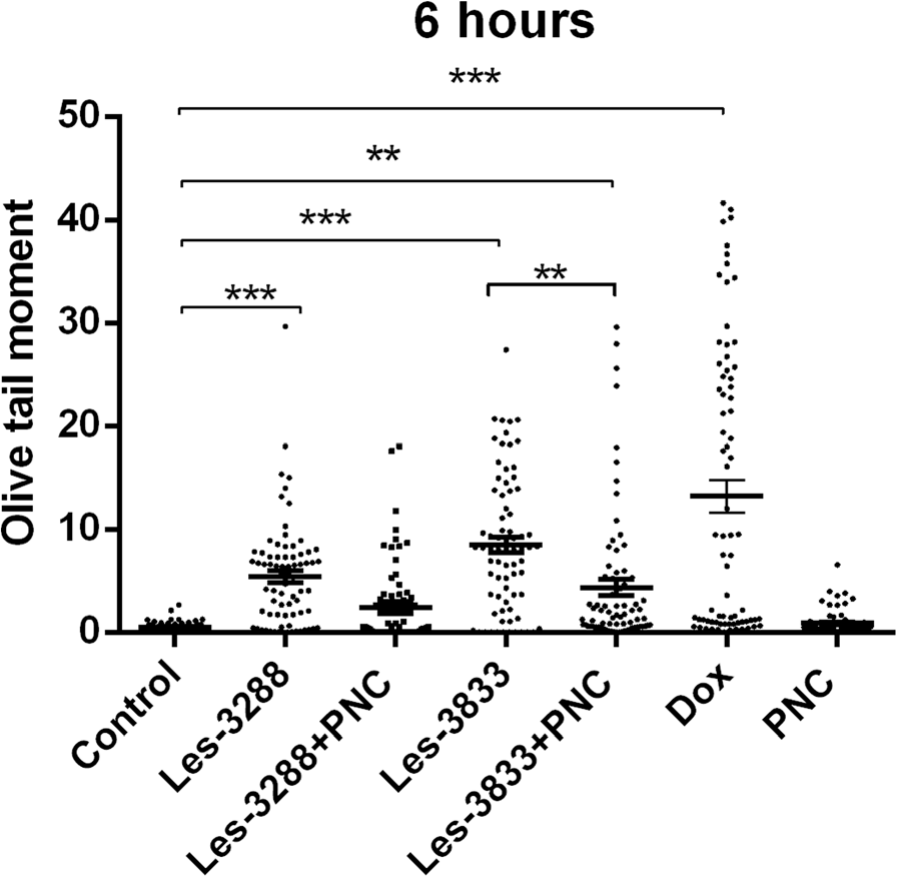
DNA damage was evaluated using the Olive tail moment in rat glioma C6 cells treated for 6 h with experimental antineoplastic compounds used at a 0.5 μg/mL concentration. *P ≤ 0.05; **P ≤ 0.01; ***P ≤ 0.001. PNC, polymeric nanocarrier; Dox, doxorubicin (positive control)

**Fig. 12:**
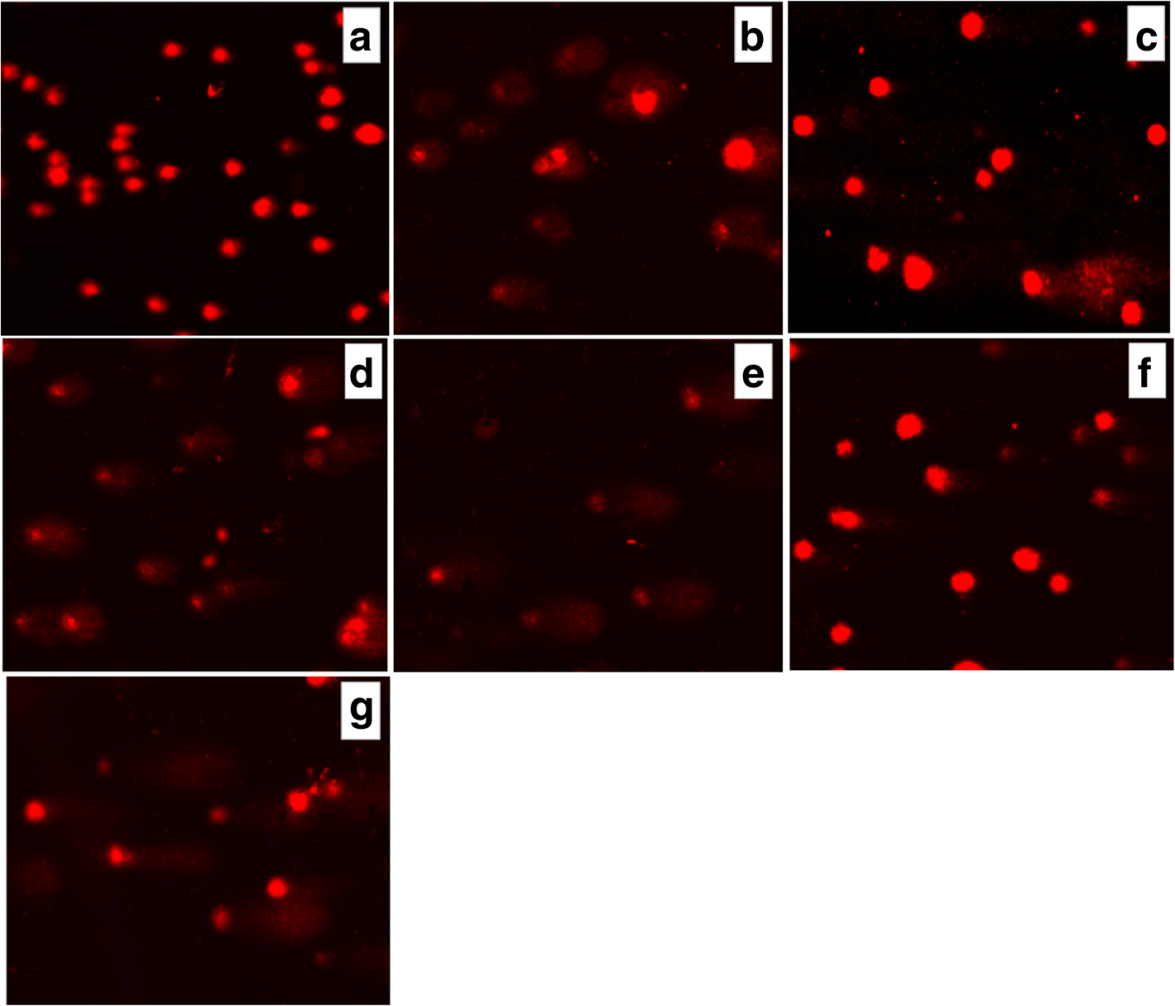
Presence of DNA in comet tail of rat glioma С6 cells after treatment for 6 h with (**a**) control (untreated cells), Les-3288 (**b**), Les-3288 + PNC (**c**), Les-3833 (**d**) Les-3833 + PNC (**e**), PNC (**f**), and doxorubicin (**g**), used at a 0.5 μg/mL concentration

An increase of cell incubation time to 6 h (Figs. 11 and 12) caused greater DNA damage in all experimental groups: Les-3288, Les-3288+PNC, Les-3833, Les-3833+PNC, and Dox (positive control). However, the effects of the Les-3288+PNC and Les-3833+PNC complexes appeared to be lower than the effects of the free forms of Les-3288 and Les-3833. It should be noted that the DNA-damaging effect of the complexes of both derivatives with the PNC was less than such effect from these derivatives used in free form.

**Fig. 13:**
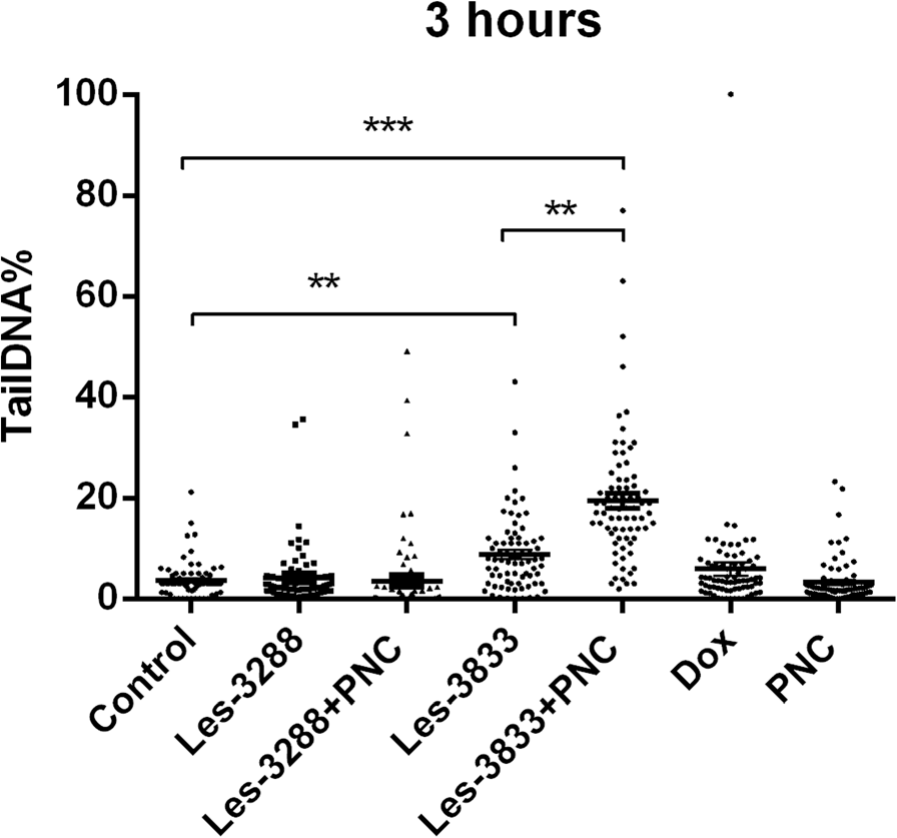
DNA damage evaluated using the % of DNA in comet tail of C6 rat glioma cells treated with experimental antineoplastic compounds during 3 h at a concentration of 0.5 μg/mL. *P ≤ 0.05; **P ≤ 0.01; ***P ≤ 0.001. PNC, polymeric nanocarrier; Dox, doxorubicin (positive control)

**Fig. 14:**
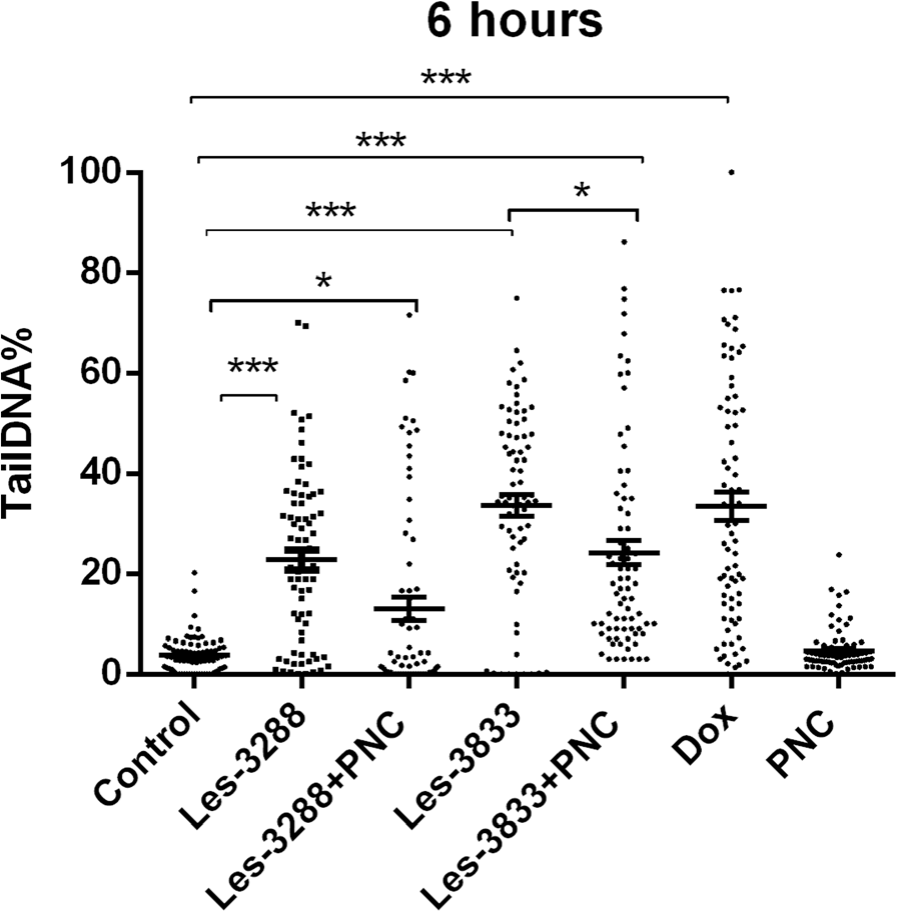
DNA damage evaluated using the % of DNA in comet tail of C6 rat glioma cells treated with experimental antineoplastic compounds during 6 h at a concentration of 0.5 μg/mL. *P ≤ 0.05; **P ≤ 0.01; ***P ≤ 0.001. PNC, polymeric nanocarrier; Dox, doxorubicin (positive control)

The results were evaluated using the mean value of the TailDNA%. The obtained data show that incubation of С6 rat glioblastoma cells with substance Les-3288, Les-3288+PNC, Les-3833, and PNC (all at concentration of 0.5 μg/mL) during 3 h (Fig. 13) does not lead to significant DNA damage (TailDNA% of 4.157 ± 0.682; TailDNA% of 3.530 ± 1,012, 8.807 ± 0.878; 3.298 ± 0.514, respectively) compared with control (TailDNA% = 3.638 ± 0.406), but cell treatment with the Les-3833+PNC complex (TailDNA% = 19.53 ± 1.48) causes more significant damage of DNA than the free form of Les-3833. An increase in the incubation time up to 6 h (Fig. 14) in all cases leads to greater damage to the DNA. Again, the level of TailDNA% detected from the action of complexes of both derivatives with the PNC was less than the level of that indicator for the action of these derivatives used in free form.

We also used an alternative, five-category classification system to classify DNA migration and calculated the DI. Table 2 summarizes the percentages of rat glioma C6 cells for each level of genotoxicity during 3 h of treatment. In the control (untreated cells), higher percentages of cells in levels 0 (81.3%) and 1 (18.6%) were detected compared to these levels in Les-3288 and PNC-treated cells. Complexation of Les-3288 with the PNC did not lead to a big change in the distribution pattern of the DNA comets. In contrast, Les-3833-treated cells showed a lower percentage of cells in 0 level (38.6%) and a higher percentage of cells in level 1 (60.0%), while Les-3833 + PNC-treated cells demonstrated a significantly lower percentage of cells in level 0 (8.0%) and much higher percentages of cells with levels 1 (76.0%), 2 (13.3%), and 3 (2.6%). The integrative indicator of DNA damage (DI) in the case of application of both Les-3288 and its complex with the PNC did not differ significantly from that for control or using free PNC, while the DI from using both Les-3833 and its complex with the PNC was even higher than the DI from using Dox.

**Table 2:** The percentages of rat glioma C6 cells for each level of genotoxicity after 3 h of treatment with the free form of compounds Les-3288 and Les-3833, and their complexes with the polymeric nanocarrier (PNC)

Sample	Comet class (% of cells)					DI
	0	1	2	3	4	
Control	81.3 ± 0.5	18.6 ± 0.4				18.60 ± 0.35
Les-3288	81.3 ± 0.3	16.0 ± 0.2	2.6 ± 0.4			21.20 ± 0.52***
Les-3288+PNC	84.0 ± 0.1	12.0 ± 0.1	2.6 ± 0.4	1.3 ± 0.2		21.10 ± 0.23**
Les-3833	38.6 ± 0.3	60.0 ± 0.5	1.4 ± 0.2			62.80 ± 0.84***
Les-3833+PNC	8.0 ± 0.3	76.0 ± 2.9	13.3 ± 0.8	2.6 ± 0.3		110.40 ± 3.13***
PNC	84.0 ± 0.6	16.0 ± 0.1				16.00 ± 0.11
Dox	62.6 ± 0.4	36.0 ± 0.4			1.3±0.17	41.20 ± 0.40***

Damage index (DI): **P ≤ 0.01, ***P < 0.001 compared with untreated control cells

During this time interval, Les-3833 + PNC-treated cells demonstrated a higher percentage of cells with level 4 (5.3%) DNA damage than cells treated with Les-3288 and the other experimental compounds, but the percentages of cells in levels 2 and 3 were lower (16.0% and 12.0%, respectively) than those from treatment with other compounds (Table 2). Table 3 summarizes the percentages of rat glioma C6 cells with DNA damage after 6 h of treatment. In general, a tendency for the appearance of DNA comets of higher classes was detected when complexes of Les-3833 and Les-3288 were used, compared with a spectrum of DNA comet classes induced by free forms of these agents (Table 3). However, the integrative indicator of DNA damage (DI) calculated for the action of complexes of Les-3833 and Les-3288 was found to be lower than such an indicator for the action of free forms of these agents. A possible explanation could be a very high DI level at 6 h action of studied agents that is even higher than such indicator found for the action of Dox (Table 3). Thus, we observed time dependence of DNA damage caused by complexes of Les-3833 and Les-3288: at 3 h of treatment, there was an increase of the DI in the case of action of Les-3833 complexes, compared to the action of the free form of Les-3833; however, at 6 h of treatment, there was a decrease in DNA damage for the action of complexes of both Les-3833 and Les-3288, compared to such damage observed for the action of the free forms of these agents. There was no significant time dependence for the action of the PNC, while a significant time-dependent (from 3 to 6 h) increase in the DI was observed for the action of Dox (from 41.2 + 0.40 to 185.2 + 0.23).

**Table 3:** The percentages of rat glioma C6 cells for each level of genotoxicity after 6 h of treatment with the free form of compounds Les-3288 and Les-3833, and their complexes with the polymeric nanocarrier (PNC)

Sample	Comet class (% of cells)					DI
	0	1	2	3	4	
Control	72.0 ± 0.5	28.0 ± 0.6				28.00 ± 0.58
Les-3288	25.3 ± 0.2	28.0 ± 0.6	37.3 ± 0.1	8.0 ± 0.3	1.3 ± 0.1	131.80 ± 0.74***
Les-3288 + PNC	62.6 ± 0.2	14.7 ± 0.9	10.7 ± 0.3	12.0 ± 0.6		72.10 ± 3.29***
Les-3833	12.0 ± 0.2	12.0 ± 0.6	41.4 ± 1.3	33.3 ± 0.2	1.3 ± 0.1	199.90 ± 2.35***
Les-3833 + PNC	10.6 ± 0.2	56.0 ± 0.3	16.0 ± 0.1	12.0 ± 0.1	5.3 ± 0.1	145.20 ± 0.85***
PNC	74.6 ± 0.9	25.3 ± 0.2				25.30 ± 0.17
Dox	12.0 ± 0.1	30.6 ± 0.4	25.3 ± 0.3	24.0 ± 0.2	8.0 ± 0.1	185.2 ± 0.23***

Damage index (DI): ***P < 0.001 compared with untreated control cells

### DNA Intercalation by 4-Thiazolidinone Derivatives—Les-3288 and Les-3833

**Fig. 15:**
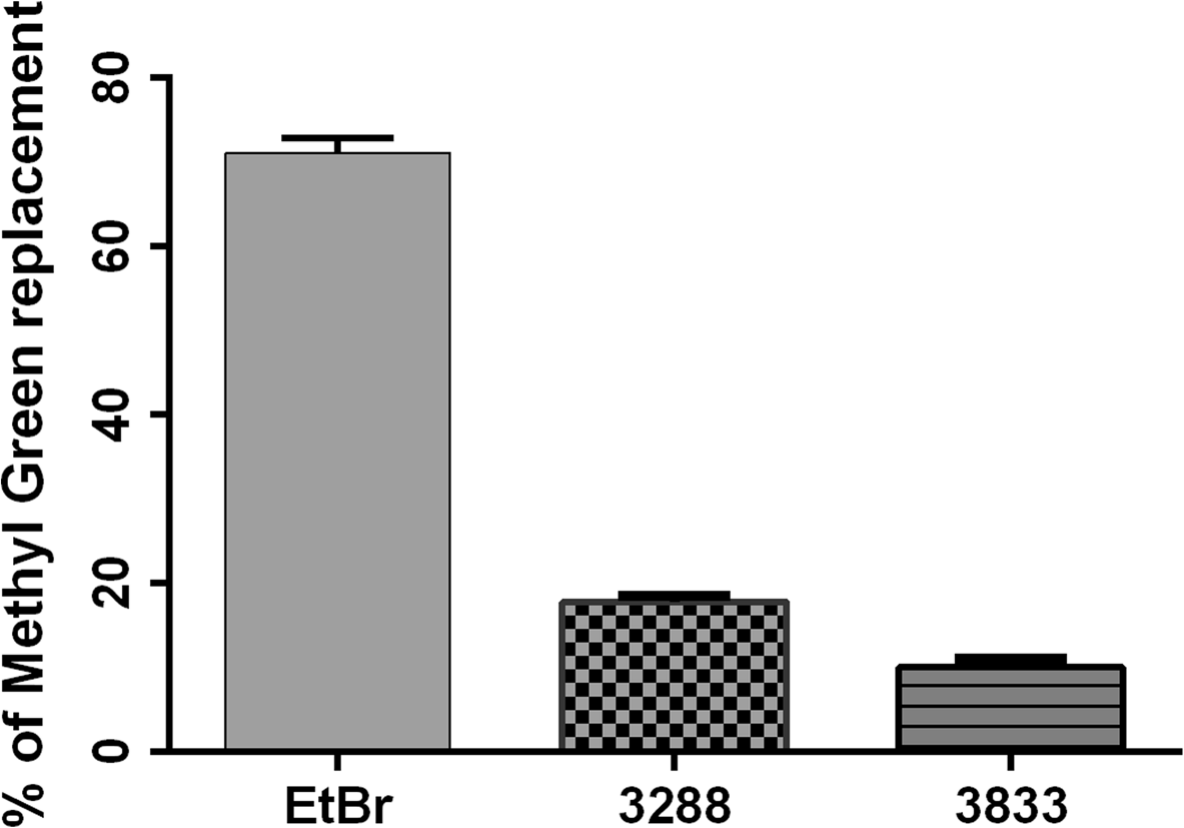
Replacement of methyl green dye intercalated into the DNA of salmon sperm by Les-3288 and Les-3833 compounds (1.0 μg/mL) and ethidium bromide (EtBr) used as a positive control

Les-3288 and Les-3833 compounds demonstrated a certain capability for intercalating DNA molecules with approximately 15% and 10%, respectively, of replacement of methyl green dye. EtBr, a well-known DNA intercalating agent, was used as a positive control, and it was shown to replace the methyl green dye much more efficiently (70%) (Fig. 15). Thus, Les-3288 and Les-3833 are not capable of effectively intercalating in between two complementary base pairs in double-stranded DNA.

## Discussion

There are two major problems that impede increasing the efficiency of treatment for cancer patients: (1) non-addressed actions of many anticancer drugs that lead to general toxicity and severe negative side effects due to damage of normal tissues and organs; (2) rapid (6–12 months) development of resistance of tumor cells to applied anticancer drugs that leads to a loss of efficacy of drug action. In addition, there is a third, technical problem of poor water solubility of many anticancer drugs. However, binding these drugs with specific carriers of the nanoscale size can help to solve the solubility problem.

In this study, we addressed the first and third of the above-mentioned problems by (a) selecting novel synthetic substances (4-thiazolidinone derivatives: Les-3288 and Les-3833) that possess both high anticancer activity and less general toxicity in tumor-bearing animals [7, 8, 23] and (b) applying a novel drug delivery platform that provides stable water-based forms of the complexes of the water-insoluble 4-thiazolidinone derivatives with a PEGylated polymer.

The 4-thiazolidinone derivatives—Les-3288 and Les-3833—are heterocyclic compounds with molecular mass of 400–600 Da. These and other related derivatives were synthesized at Lviv National Medical University (Ukraine), and most of them have been tested under the Developmental Therapeutic Program at the National Cancer Institute in Bethesda, MA, (USA) [35–38]. Based on these evaluations of their antineoplastic activity, the most promising substances were selected for further study of the mechanisms of their cytotoxic action and anticancer effects [25]. Les-3833 demonstrated high toxicity towards B16F10, WM793, and SK-Mel-28 melanoma cells, and against human lung A549, breast MCF-7, colon HCT116, and ovarian SKOV3 cancer cells and leukemia cells (L1210, Jurkat, HL-60 lines) [23, 25, 39]. Les-3288 and Les-3833 showed high toxicity against mammalian glioma cells (C6, U251, U373 lines) [23, 24].

Earlier, we found that Les-3288 and Les-3833 were the most toxic among the other studied 4-thiazolidinone derivatives towards rat glioma C6 cells and human glioblastoma U251 cells [23, 24], although their application for cell treatment was very complicated due to their absolute insolubility in water. Only using DMSO with additional heating was effective for preparing a soluble form of the derivatives (see the “Materials and Methods” section). Thus, it was reasonable to search for a way of improving the delivery of these derivatives to target cells, and we used a synthetic PNC to accomplish this task. The complexation of the studied derivatives of 4-thiazolidinone with the applied PNC also enhanced the effectiveness of their antineoplastic action. The mechanisms of the pro-apoptotic action of these compounds were demonstrated using western blot and FACS analyses [23, 24]. It should be noted that in healthy experimental animals, Les-3288 and Les-3833 produce much fewer negative side effects such as cardiotoxicity [40], hepatotoxicity [8], and nephrotoxicity [7] than the widely used anticancer chemotherapeutic agent Dox.

In a previous study, we showed that Les-3288 did not induce ROS production during its pro-apoptotic action in vitro and effects in experimental laboratory animals [23], while the action of Les-3833 led to such production; however, it was less than that of Dox. ROS are considered to be the main effectors of negative side effects of anticancer drugs, particularly Dox [41, 42]. Thus, the Les-3288 and Les-3833 compounds could be prospective anticancer drugs because they possess antitumor activity, but do not demonstrate general toxicity at the level characteristic for effective anticancer medicines such as Dox.

A great impediment to promoting Les-3288 and Les-3833 as anticancer drugs is their poor water solubility. This problem also exists for other anticancer medicines, such as taxols [2, 4]. For paclitaxel, this problem was solved by using a special oil for preparing the medicinal formulation [2, 4, 43]. However, specific delivery platforms are considered to be more promising for creating “smart” drugs that possess effective action in the organism [3, 13]. Specific functional polymer surfactants with block, comb-like, and block/branched architectures, including PEG-containing ones, as well as derived water forms have been developed and proposed for application as carriers for the delivery of Dox [26, 27] and the metal chemotherapeutic agent KP-1019 [10] at the Department of Organic Chemistry of Lviv Polytechnic National University.

Since the 4-thiazolidinone derivatives are water-insoluble compounds, DMSO was used to prepare their water-soluble forms. To avoid the negative consequences of using a toxic solvent like DMSO, we complexed these derivatives with the above-noted PNC. Highly dispersed and stable nanoscale water dispersions of these substances were obtained and applied for treatment of rat glioma C6 cells.

The use of complexes of 4-thiazolidinone derivatives with the PNC led to reduced general toxicity of these compounds in experimental animals. Earlier, we found that when experimental antitumor agents (Les-3288, Les-3833, Les-3882) were applied in a complex with a synthetic polymeric carrier, their action was accompanied by much smaller changes in the biochemical parameters in blood serum that are characteristic for cardiotoxicity [40], hepatotoxicity [8], and nephrotoxicity [7], compared to those for Dox. Thus, complexation of these antitumor agents with the PNC and their application in the form of water-soluble stable delivery systems reduces their toxic effects in experimental animals, compared with the action of these substances in a free form [44]. Several systems for paclitaxel delivery have been proposed including polymeric nanoparticles, lipid-based formulations, polymer conjugates, inorganic nanoparticles, carbon nanotubes, nanocrystals, and cyclodextrin nanoparticles [43].

Thus, complexation of Les-3288 with the PNC significantly increased the pro-apoptotic activity of this experimental drug, thereby allowing us to advance it to pre-clinical studies on animal tumor models. Complexation of Les-3833 with the same type of nanocarrier was less efficient, and thus another type of polymer should be used.

The developed water-based forms of the complexes, as well as the free derivatives, were applied for treatment of rat glioma C6 cells. It was found that these water-based forms of the complexes of the PNC with the 4-thiazolidinone derivatives—Les-3288 and Les-3833—enhanced the pro-apoptotic action of these compounds. In another study, we demonstrated that the induction of apoptosis by Les-3288 was not accompanied by ROS induction, opposite to the action of Dox and Les-3833 [23]. Thus, high antitumor activity of Les-3288 together with its low general toxicity when targeting the malignancy in NK/Ly lymphoma-bearing mice suggest great potential for this experimental anticancer compound. The presented data also predict the potential usefulness of Les-3288 and Les-3833 compounds complexed with the PNC for glioma treatment.

Cell division, differentiation, and death are principal physiological processes that regulate tissue homeostasis in multicellular organisms. A disruption of genomic integrity and impaired regulation of cell death may both lead to uncontrolled cell growth. A compromised cell death process can also promote genomic instability. It is becoming clear that dysregulation of the cell cycle and cell death processes plays a pivotal role in the development of major disorders such as cancer, cardiovascular disease, infection, inflammation, and neurodegenerative diseases [45].

We have found that some of the 4-thiazolidinone derivatives, namely, Les-3288 and Les-3833, were even more effective than Dox in the induction of apoptosis in rat C6 glioma and human U251 glioblastoma cells in vitro [23, 24]. It should be noted that brain tumors belong to malignancies that are the most resistant to applied chemotherapies, and survival rates in patients with these tumors are extremely low [44].

It has been shown that the complexation with this PNC enhanced the pro-apoptotic action of 4-thiazolidinone derivatives (Les-3288 and Les-3833) [44]. In another study [23], we demonstrated that the induction of apoptosis by Les-3288 was not accompanied by ROS induction, opposite to the action of Dox and partially opposite to that of Les-3833. These data could explain the lower general toxicity of Les-3288 compared with that of Dox [23, 24].

The results of FACS analysis are in agreement with the suggestion that an enhancement of the pro-apoptotic action of the studied 4-thiazolidinone derivatives is due to their complexation with a novel PNC. Such complexation of Les-3288 and Les-3833 increased the ratio of pre-G1 cells in the population of treated rat glioma C6 cells, decreased the ratio of G1 cells, and increased the action of G2/M cells. These data suggest an enhancement of both cytotoxic action (apoptosis) and cytostatic action (block in G2/M phase of cell cycle) of the studied derivatives complexed with the PNC. In general, the apoptosis mechanism of glioma cell killing by the Les-3288 + PNC complex and the Les-3833 + PNC complex was demonstrated by the results of FACS analysis of the appearance of Annexin V single-positive rat glioma C6 cells.

Involvement of the apoptosis mechanisms in the action of the studied 4-thiazolidinone derivatives was also confirmed by the results of the DNA comet assay. It was found that the complexation of Les-3833 with the PNC led to a decrease (compared to free Les-3833) in the ratio of rat glioma C6 cells in “0” DNA comets and sіmultaneous increase in the ratio of “1,” “2,” and “3” comets (3-h treatment of cells). At 6 h of treatment by the Les-3833+PNC complex, there was a large increase (compared to free Les-3833) in the ratio of “1” comets, an increase in the ratio of cells with the most expressed DNA damage (“4” comets), and a decrease in the ratios of “2” and “3” comets. It should be noted that the DNA damaging effects of Les-3288 and Les-3833 measured at 6 h was higher than such effects of complexes of these derivatives with the PNC (see Table 3). A possible explanation here could be a very high level of the DI at 6 h action of studied agents that is even higher than the level of that indicator found for the action of Dox (Table 3). There was no time dependence in the action of the PNC, while a significant time-dependent (from 3 to 6 h) increase (from 41.2 + 0.40 to 185.2 + 0.23) in the DI was detected for the action of Dox.

Thus, a complexation of 4-thiazolidinone derivatives—Les-3288 and Les-3833—with a PNC enhanced the cytotoxic effect of these compounds towards rat glioma C6 cells, and that effect was achieved through the apoptosis mechanisms including DNA damage observed as DNA comets in the electrophoresed cells.

We used the DNA intercalation test to investigate the direct targeting of DNA by the 4-thiazolidinone derivatives and revealed only weak replacement of methyl green dye (15% and 10% replacement for Les-3288 and Les-3833, respectively) that intercalated into the DNA of salmon sperm, compared to the replacement by EtBr, used as a positive control (70%).

Thus, conducting the cytotoxicity experiments in vitro with the 4-thiazolidinone derivatives—Les-3288 and Les-3833—was more convenient due to using the water-based forms of the 4-thiazolidinone derivatives—Les-3288 and Les-3833—complexed with the novel PNC. Besides, we have demonstrated that the novel synthetic 4-thiazolidinone derivatives (Les-3833 and Les-3288) possessed treatment effects *i*n vivo towards NK/Ly lymphoma grafted to BALB/C mice, and those effects were comparable to such effects of Dox. At the same time, the action of these derivatives led to much fewer negative side effects measured as changes in the number of erythrocytes, neutrophils, and lymphocytes in the animals’ blood and the activity of aspartate and alanine aminotransferases in their blood serum, compared with the action of Dox.

## Conclusions

The complexation of Les-3288 and Les-3833 with the PEG-containing PNC enhanced their cytotoxic action towards rat glioma C6 cells. The cytotoxic action was realized by apoptotic mechanisms and confirmed by FACS analysis as the presence of the pre-G1 fraction in the rat glioma C6 cells and of Annexin V-single-positive cells. DNA comet analysis revealed single-strand brakes in the nuclear DNA of treated glioma C6 cells that probably was not caused by the intercalation of the studied compounds into the DNA molecule. The Les-3288 and Les-3833 stabilized by the amphiphilic PEG-containing PNC resulted in the water-based forms and provided enhancement of their antitumor effect in vitro in comparison with the free form of the drugs.

## Abbreviations

DMSO: Dimethylsulfoxide
Dox: Doxorubicin
EtBr: Ethidium bromide
FACS: Fluorescence-activated cell sorting
ROS: Reactive oxygen species
PBS: Phosphate buffered saline
PEG: Polyethylene glycol
PI: Propidium iodide
PNC: Polymeric nanocarrier
SEM: Scanning electron microscopy
TEM: Transmission electron microscopy
VEP: 2-Tret-butylperoxy-2-methyl-5-hexen-3-in
